# Identification of blood-feeding sources in *Panstrongylus*, *Psammolestes*, *Rhodnius* and *Triatoma* using amplicon-based next-generation sequencing

**DOI:** 10.1186/s13071-020-04310-z

**Published:** 2020-08-31

**Authors:** Luisa M. Arias-Giraldo, Marina Muñoz, Carolina Hernández, Giovanny Herrera, Natalia Velásquez-Ortiz, Omar Cantillo-Barraza, Plutarco Urbano, Andrés Cuervo, Juan David Ramírez

**Affiliations:** 1 Grupo de Investigaciones Microbiológicas-UR (GIMUR), Departamento de Biología, Facultad de Ciencias Naturales, Universidad del Rosario, Bogotá, Colombia; 2 Grupo de Biología y Control de Enfermedades Infecciosas, Universidad de Antioquia, Medellín, Colombia; 3 Grupo de Investigaciones Biológicas de la Orinoquia, Fundación Universitaria Internacional del Trópico Americano (Unitropico), Yopal, Colombia; 4 Secretaría Departamental de Salud de Arauca, Arauca, Colombia

**Keywords:** Chagas disease, *Trypanosoma cruzi*, Triatominae, Feeding sources, NGS, Colombia

## Abstract

**Background:**

Triatomines are hematophagous insects that play an important role as vectors of *Trypanosoma cruzi*, the causative agent of Chagas disease. These insects have adapted to multiple blood-feeding sources that can affect relevant aspects of their life-cycle and interactions, thereby influencing parasitic transmission dynamics. We conducted a characterization of the feeding sources of individuals from the primary circulating triatomine genera in Colombia using amplicon-based next-generation sequencing (NGS).

**Methods:**

We used 42 triatomines collected in different departments of Colombia. DNA was extracted from the gut. The presence of *T. cruzi* was identified using real-time PCR, and discrete typing units (DTUs) were determined by conventional PCR. For blood-feeding source identification, PCR products of the vertebrate *12S* rRNA gene were obtained and sequenced by next-generation sequencing (NGS). Blood-meal sources were inferred using blastn against a curated reference dataset containing the *12S* rRNA sequences belonging to vertebrates with a distribution in South America that represent a potential feeding source for triatomine bugs. Mean and median comparison tests were performed to evaluate differences in triatomine blood-feeding sources, infection state, and geographical regions. Lastly, the inverse Simpsonʼs diversity index was calculated.

**Results:**

The overall frequency of *T. cruzi* infection was 83.3%. TcI was found as the most predominant DTU (65.7%). A total of 67 feeding sources were detected from the analyses of approximately 7 million reads. The predominant feeding source found was *Homo sapiens* (76.8%), followed by birds (10.5%), artiodactyls (4.4%), and non-human primates (3.9%). There were differences among numerous feeding sources of triatomines of different species. The diversity of feeding sources also differed depending on the presence of *T. cruzi*.

**Conclusions:**

To the best of our knowledge, this is the first study to employ amplicon-based NGS of the *12S* rRNA gene to depict blood-feeding sources of multiple triatomine species collected in different regions of Colombia. Our findings report a striking read diversity that has not been reported previously. This is a powerful approach to unravel transmission dynamics at microgeographical levels.
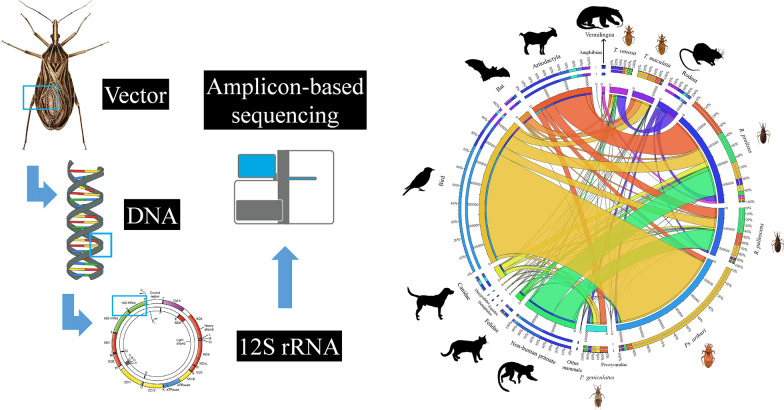

## Background

Triatomines (Hemiptera: Reduviidae) are hematophagous insects that play an important role as vectors of *Trypanosoma cruzi*, the causative agent of Chagas disease [1], which is a neglected tropical disease (NTD). Over 8 million people are considered infected with *T. cruzi*, and more than 200,000 new cases are identified each year [2, 3]. The parasite *T. cruzi* boasts tremendous genetic diversity and has been divided into six discrete typing units (DTUs) from TcI to TcVI [4], which are associated with various clinical manifestations, geographical distribution, and ecotopes [5]. This difference in ecotopes results in the ability of invading both “domestic” and “sylvatic” environments, which is facilitated by its vectors, that have adapted to multiple blood-feeding sources [6], including various vertebrates, such as rodents, humans, non-human primates, bats, marsupials, dogs, armadillos, porcupines, cows, goats and birds. This has been reported over the years *via* scientific studies using Sanger sequencing techniques [7–10].

Feeding habits can affect relevant aspects of insect life-cycles and interactions. For example, cellulase activity within digestion is affected by the feeding habits of termites [11]; in addition, infection with *Mycobacterium ulcerans* is conditioned by the feeding behavior of water bugs due to a possible symbiotic relationship between the host and insect [12]. Moreover, the bacterial and fungal communities in the gut of certain insects are defined by their feeding habits [9, 13], which can affect the effectivity of insects as vectors, as has been shown for *Anopheles* in previous studies [14–20]. Due to the aforementioned effects, these variables have an impact on transmission dynamics [21, 22], which makes knowledge of feeding habits important for the development of effective prevention and control strategies for tropical diseases.

In Colombia, 15 triatomine species are found to be naturally infected with *T. cruzi*, with *Rhodnius prolixus*, *Triatoma dimidiata* and *Panstrongylus geniculatus* being the most relevant [23]. The infection rate within these triatomines can surpass 40% [8, 24–27], making it higher than that within other triatomines [28, 29], and explains the relevance of these particular triatomine species, along with their capability of colonizing human dwellings [23]. The feeding preferences of these insects include humans; arboreal mammals, such as New World monkeys, sloths, opossums, and coatis; domestic mammals, such as cats, dogs, cows and rodents, as well as other animals such as reptiles and bats [7–10, 30].

Latter studies have explored the feeding habits and interactions of triatomines, such as the work by Dumonteil et al. [9], in which triatomines were observed simultaneously feeding on different vertebrates. These authors also constructed a possible transmission network for the parasite, involving the 14 vertebrate hosts elucidated in this study [9]. Recently, Erazo et al. [27] identified 18 vertebrate species as a feeding source for *R. prolixus*. According to this study, the infection rate varied among triatomines feeding upon different vertebrates in a way suggesting that diet specialization plays a pivotal role in defining the transmission dynamics of Chagas disease. Although the described range of triatomine feeding sources is wide [7, 8, 10, 22, 31] and is known to affect crucial aspects of their life-cycles and interactions, this aspect has not been vastly evaluated in order to completely understand the dynamics involved. Furthermore, only a few studies have been conducted using next-generation sequencing (NGS), particularly amplicon-based sequencing [9, 32, 33], despite its capacity to reveal multiple host species simultaneously and characterize many more samples than traditional techniques [32].

Depicting the complexity of feeding preferences among triatomine bugs is of pivotal importance for building efficient control strategies for these vectors, given that these preferences can define the behavior and explain the presence of the insects under certain conditions (i.e. modify parasite transmission routes). Ultimately, all this information could be important for completely understanding the Chagas disease transmission and potentially improving the current measures established against it. For this, it is also necessary to assess the *T. cruzi* presence in the vector, alongside the characteristics of the parasite, such as its genetic diversity. Therefore, we herein conducted a robust characterization of feeding sources using amplicon-based NGS from available individuals of the primary triatomine genera found in Colombia (*Panstrongylus*, *Rhodnius* and *Triatoma*) and included *Psammolestes* due to its recent evidence of *T. cruzi* infection. This study was also complemented with detecting *T. cruzi* infection and assessing the genetic diversity of *T. cruzi*.

## Methods

### Insect sampling, dissection and DNA extraction

**Fig. 1:**
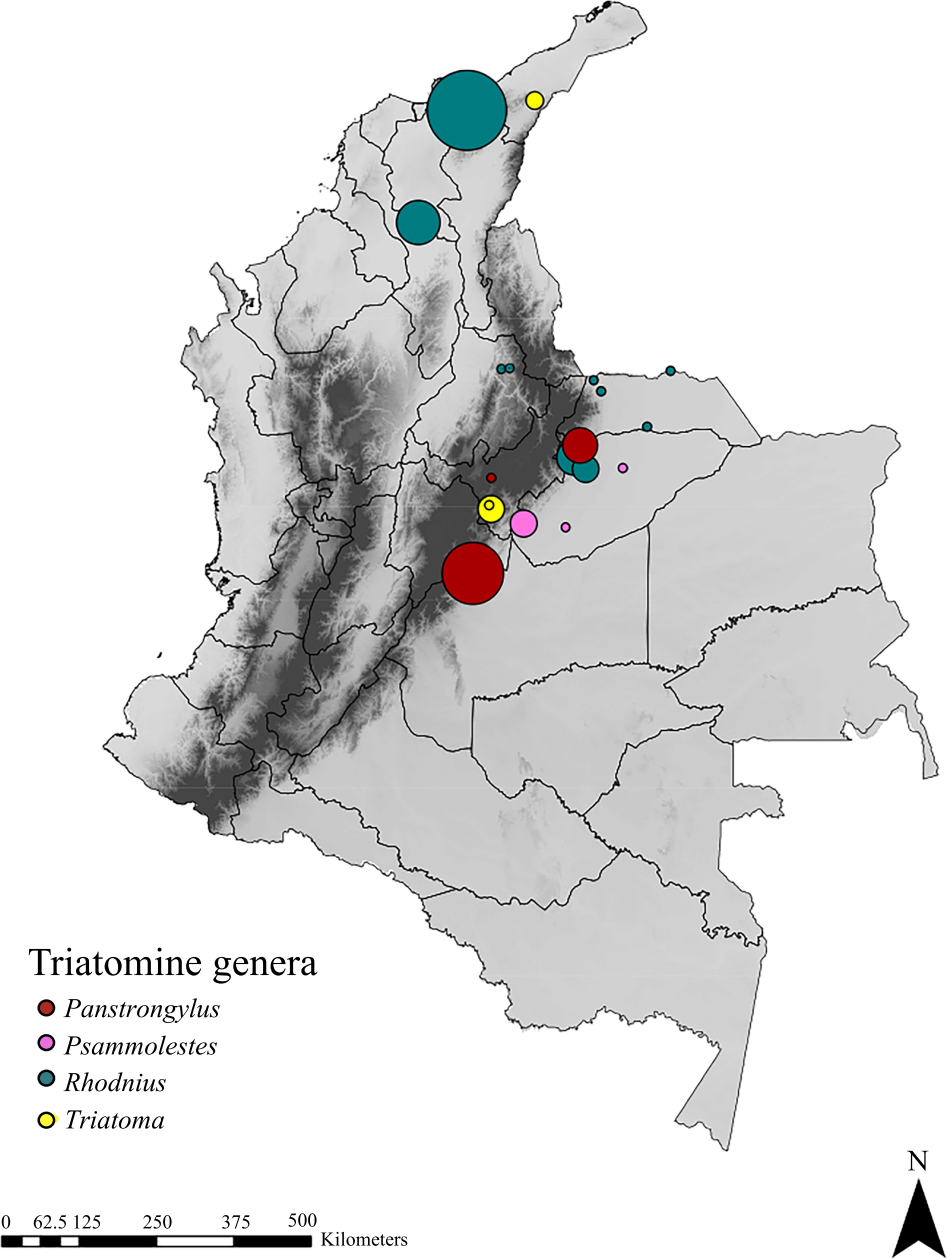
Geographical distribution of the 42 triatomine samples used in this study. Circle size is proportional to the number of triatomines

Forty-two triatomines (see Additional file 1: Table S1) collected between 2012 and 2018 in different districts of Colombia (Arauca, Bolívar, Boyacá, Casanare, La Guajira, Magdalena, Meta and Santander) were used in this study (Fig. 1). These specimens were collected in the framework of previous studies using different entomological surveillance techniques for each ecotope (i.e. domestic, peridomestic and sylvatic) as described elsewhere [34]. In total, the triatomines used consisted of 6 *P. arthuri*, 15 *R. prolixus*, 7 *R. pallescens*, 8 *P. geniculatus*, 3 *T. maculata* and 3 *T. venosa.* Manipulation of triatomine individuals was carried out, taking into account the field permit from Autoridad Nacional de Licencias Ambientales (ANLA) 63257-2014 provided by Universidad del Rosario. The collection of all triatomines was conducted on public land. Insects were stored in Eppendorf tubes with 100% ethanol and, upon arrival at the laboratory, were frozen at − 20 °C until dissection. The abdominal region was excised and washed 3 times with ultra-pure water in preparation for posterior use. DNA from the gut was extracted using a DNeasy Blood and Tissue Kit (Qiagen, Hilden, Germany), and DNA concentrations were determined using a NanoDrop ND-100 spectrophotometer (Thermo Fisher Scientific Inc., Waltham, MA, USA).

### Detection and genotyping of *T. cruzi*

The presence of *T. cruzi* parasites within triatomines was detected using real-time PCR with the primers Cruzi1 (5′-AST CGG CTG ATC GTT TTC GA-3′) and Cruzi2 (5′-AAT TCC TCC AAG CAG CGG ATA-3′), as well as probe Cruzi3 (5′-CAC ACA CTG GAC ACC AA-3′), as described elsewhere [8, 35]. Samples were considered positive when the amplification exceeded the threshold of fluorescence of 0.01. For insects yielding positive results by initial qPCR, it was necessary to discriminate if detection was due to the presence of *T. cruzi* or *Trypanosoma rangeli*, another trypanosome species circulating in the Neotropics transmitted by triatomine bugs, which do not have a pathogenic effect over its mammalian hosts [36], and therefore, a kinetoplast fragment DNA amplification was performed using primers 121 (5′-AAA TAA TGT ACG GGK GAG ATG CAT GA-3′) and 122 (5′-GGT TCG ATT GGG GTT GGT GTA ATA TA-3′) as described elsewhere [37]. For insects identified as being positive for *T. cruzi*, TcI and non-TcI DTUs were discriminated, *via* the usage of part (PCR directed to the SL-IR region only) of the algorithm implemented by Hernández et al. [8]. Finally, for the TcI-positive samples, we discriminated between TcIDom and TcISylv, also adopting part of the algorithm used by Hernández et al. [8].

### Feeding sources

PCR products of the vertebrate *12S* rRNA gene were obtained through amplification of a 215-bp fragment using primers L1085 (5′-CCC AAA CTG GGA TTA GAT ACC C-3′) and H1259 (5′-GTT TGC TGA AGA TGG CGG TA-3′) [9]. These fragments were pooled for sequencing after independent library construction, and 2 × 300 paired-end sequencing was performed on an Illumina HiSeq flow cell (Illumina, San Diego, USA). Amplicon sequencing was conducted by Novogene (Beijing, China).

The *12S* rRNA sequences produced by the Illumina HiSeq went through a quality control (QC) step applied with aim of reducing the technical bias (PCR or sequencing related) and to assure that the diversity detected truthfully reflected the biological scenario. During this QC step, sequences with incongruences in the barcode or without the correct primer sequence as well as short (< 200 bp in length) and low quality (with a minimal average quality score of 25) reads were discarded. The less frequent sequences were not removed in order to capture possible rare food sources [33]. The quality filtering was made using QIIME software [38]. The high-quality sequences were used to describe the feeding source preferences of the triatomines. Blood meals were inferred using BLASTn against a curated dataset (see ‘Reference dataset construction’ below), considering a minimum of 95% identity and an e-value of 10 as a match. The 5 best vertebrate matches for each read were narrowed down to select the best result per read selecting the highest identity percentage and the lowest e-value. Matches with different vertebrate species with the same similarity were detected with a reduced number of reads (0.87% on average); these data were excluded for subsequent analyses given their ambiguity.

The number of reads corresponding to each vertebrate in the reference dataset was recorded and used as a proxy of its abundance within the triatomine diet as reported elsewhere [9]. Additionally, we used the online tool CIRCOS (http://circos.ca/) to graphically represent the relative abundance and distribution of feeding sources for all triatomines per species [39].

### Reference dataset construction

To build this database, we considered all the sequences contained in the NCBI Nucleotide database (https://www.ncbi.nlm.nih.gov/nuccore/). For this, we conducted an advanced search where “*12S* rRNA” was targeted as the gene name and “vertebrates” was targeted as the organism. The geographical distribution of each non-human species was checked online to select the vertebrates present in Latin America (Mexico, Central America and South America) taking into account the dispersion capacity of numerous vertebrates and the consequent potential of their presence in the regions where we collected the insects. The only vertebrates excluded from this should be the ones with biological restrictions that limit their distribution (e.g. endemic species in regions different from Latin America). Therefore, for the geographical authentication we accessed the following major public repositories: https://www.fishbase.in for fish; https://bioweb.bio/faunaweb/mammaliaweb/ for mammals; http://reptile-database.reptarium.cz for reptiles; http://amphibiaweb.org/ for amphibians; https://www.hbw.com/ for birds; and https://www.iucnredlist.org/ and https://www.naturalista.mx/ for any of the above (in case the information was not available in the previous databases); where available maps and literature information about the distribution of each vertebrate were used. The geographical distribution of each non-human species did not have to be limited to Latin America, introduced species were also considered, and the *Homo sapiens* sequence with GenBank accession number X62996.1 was included without taking the geographical factor into consideration. The length and pertinence of all the sequences used in this reference file were double-checked applying the search criteria by different members of the team, and with an additional step where BLAST was executed against the reference dataset using all the *H. sapiens* sequences available in the NCBI, to verify the absence of wrongly assigned sequences. Throughout the whole process, 3851 sequences were initially evaluated and a total of 397 definitive sequences comprised our reference FASTA file (Additional file 2: Alignment S1).

### Statistical analyses

Median and mean comparison tests, according to the normality of the data, were implemented to evaluate differences within blood-feeding sources among triatomines in terms of (i) the reads of each vertebrate category within individual triatomine species, (ii) the alpha diversity index calculated for each triatomine species, and (iii) the *T. cruzi* infection state. These analyses were conducted using the R software version 3.6.1, fixing a 0.05 significance level for all hypothesis tests. The normality of data was verified implementing a Shapiro-Wilk normality test. When normality was met and the comparison evaluated was of multiple grouping (e.g. triatomine species), mean values were compared using ANOVA, and when normality was not met, median values were compared implementing the Kruskal-Wallis chi-square test. When normality was met and the comparison evaluated was between 2 groups (i.e. *T. cruzi* infection state), mean values were compared using the Welch two sample t-test, and for the opposite cases, the Wilcoxon test was used to compare median values. When necessary, individual t-tests were performed to further explore the statistical differences detected. All this was performed using R Commander (Rcmdr) [40]. Alpha diversity was estimated with the inverse Simpson diversity index, which was calculated for each triatomine species using the same software.

## Results

### Detection and genotyping of *T. cruzi*

The overall *T. cruzi* infection frequency was 83.3% (*n* = 35). The frequency of *T. cruzi* infection within *P. geniculatus* and *R. prolixus* was 87.5% (7/8) and 73.3% (11/15), respectively. In the case of *Ps. arthuri*, *R. pallescens*, *T. maculata* and *T. venosa*, the frequency of infection was 100%. No insects were positive for *T. rangeli.*

TcI and TcII–TcVI were detected with TcI being the predominant DTU (80%, *n *= 28/35), taking into account that mixed cases are also considered as positive for TcI (Additional file 1: Table S1). Among the positive samples for this DTU, we found 7 *P. geniculatus* (25%), 4 *Ps. arthuri* (14.3%), 6 *R. pallescens* (21.4%), 7 *R. prolixus* (25%), 1 *T. maculata* (3.6%) and 3 *T. venosa* (10.7%). The only case of TcII–TcVI found alone (i.e. not within a mixed infection case) (2.9%, *n* = 1/35) belonged to a single *T. maculata*. One additional category was established for those individuals in which the DTU could not be determined as there was either inadequate DNA available for PCR or, despite the result being *T. cruzi*-positive, the parasitic load appeared too low to be identified. In this case, we found 6 ND (not detected) cases (17.1%, *n* = 6/35) which corresponded to 1 *R. pallescens*, 4 *R. prolixus* and 1 *T. maculata*. Regarding the previously mentioned mixed infection (TcI + TcII–TcVI), we identified 5 cases (14.3%, *n* = 5/35) corresponding to 3 *P. geniculatus*, 1 *Ps. arthuri* and 1 *R. prolixus*.

Within the cases detected as positive for TcI, TcIDom and TcISylv were also detected, with 9 (32.1%, *n* = 9/28) found to be TcIDom, of which 3 corresponded to *P. geniculatus*, 1 to *Ps. arthuri*, 3 to *R. pallescens*, 1 to *R. prolixus* and 1 to *T. venosa* (Additional file 1: Table S1). In addition, 5 cases were detected as positive for TcISylv (17.9%, *n* = 5/28), where 3 belonged to *R. prolixus* samples and 2 belonged to *P. geniculatus*. We also detected 5 (17.9%, *n *= 5/28) mixed infection cases in which TcIDom and TcISylv are found together within a single sample; these consisted of 1 *P. geniculatus*, 3 *Ps. arthuri* and 1 *R. prolixus*. There were 9 ND cases (32.1%, *n* = 9/28) corresponding to 1 *P. geniculatus*, 3 *R. pallescens*, 2 *R. prolixus*, 1 *T. maculata* and 2 *T. venosa*.

### Feeding sources identification

A total of 67 feeding sources were detected within the 42 collected insects as a result of analyses of approximately 7 million total reads. The predominant feeding source was found to be *H. sapiens* (76.8%), followed by birds (10.5%), artiodactyls (4.4%), and non-human primates (3.9%) (Additional file 3: Figure S1). The totality of detected vertebrate species is presented in Additional file 4: Figure S2. These species were arbitrarily grouped to facilitate their graphic display (shown in Additional file 5: Table S2). This grouping aimed to maintain a maximum of 15 categories, therefore, species were grouped by family, order, or broad range (i.e. bats and birds) and the category “Other mammals” contained vertebrates that did not share one of the previous taxonomic categories with any other species.

**Fig. 2:**
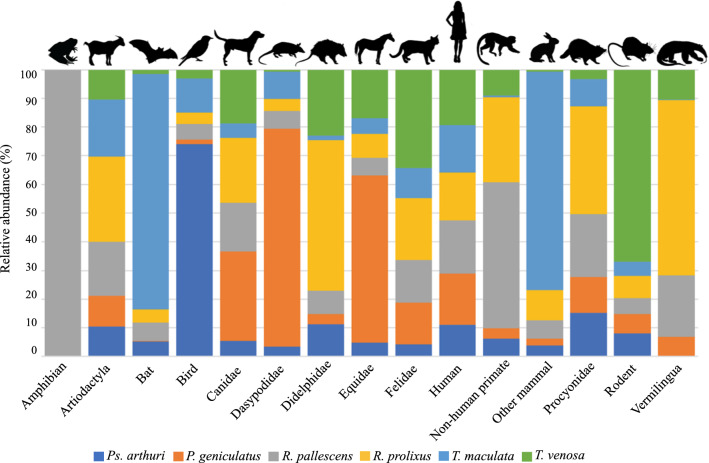
Number of reads found for each vertebrate group detected. Bar colors represent the triatomine species in which these reads were found. The total number of reads was 7,178,645

**Fig. 3:**
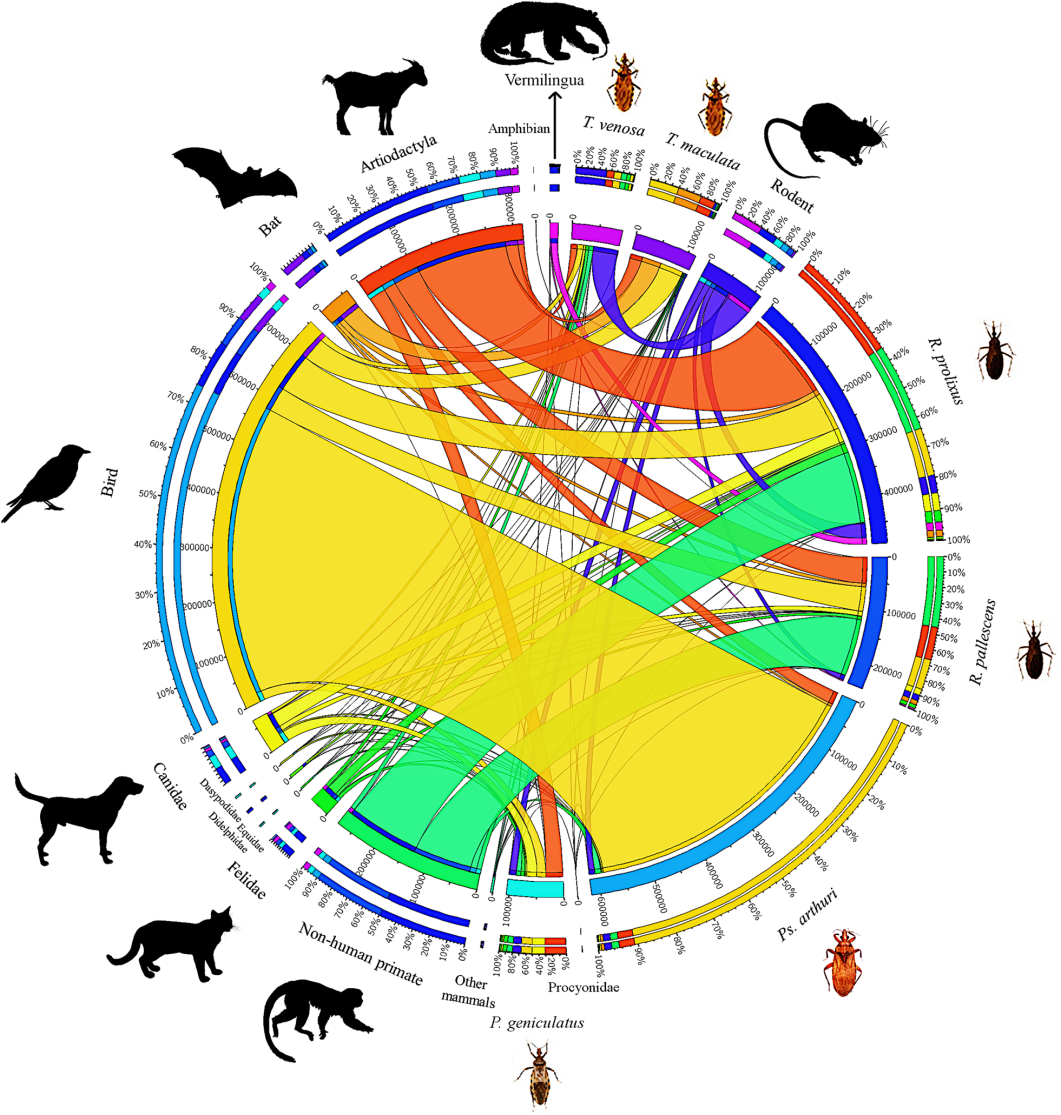
Circular web made with CIRCOS online tool representing the relative abundance of the non-human feeding sources detected in each of the evaluated triatomine species. Vertebrate feeding sources are conveniently shown in the arbitrary grouping established for this study. Since humans are not shown, the total number of reads here was 1,662,701

We found that, despite all the collected triatomine species fed on almost every group of vertebrate detected, they did it in apparent different proportions (Figs. 2, 3). While the reads belonging to *H. sapiens* seem to be equally present in every triatomine species, this is the only case where it is as clear. For instance, and without considering *H. sapiens*, we observed that *Ps. arthuri* seems to feed mostly on birds, while *T. maculata* has the highest proportion of reads belonging to bats. Also, *T. venosa* was the species for which the highest number of reads corresponding to rodents was found, and more than 50% of reads corresponding to anteaters (Vermilingua) were found in *R. prolixus*. It is also worth noting than more than 80% of reads corresponding to non-human primates were found in *R. pallescens* and *R. prolixus* together. Lastly, despite the low number of reads, the totality of amphibian reads was found in *R. pallescens* bugs (Fig. 2). These preferences were graphically represented by the CIRCOS plot containing read frequencies of the vertebrate hosts detected within each triatomine genus (Fig. 3), where *H. sapiens* was not plotted given its predominance and homogeneous distribution among triatomine species. The CIRCOS plot showed that more than 50% of the reads identified as sequences belonging to artiodactyls showed an association with *R. prolixus*, and all reads corresponding to amphibians showed an association with *R. pallescens* (Fig. 3).

In terms of vertebrate species, different proportions were found in each triatomine species, and only some of these were detected in all triatomine species. A more detailed summary of the vertebrate host species and their relative abundances within triatomines is shown in Additional file 4: Figure S2, where we found that *Eudromia elegans* and *Numida meleagris* were observed only in *Ps. arthuri*; *Didelphis albiventris*, *Philander opossum* and *Telmatobius* sp. only in *R. pallescens*; and *Chironectes minimus*, *Coccyzus americanus*, *Sciurus flammifer* and *Tremarctos ornatus* only in *R. prolixus* bugs.

**Fig. 4:**
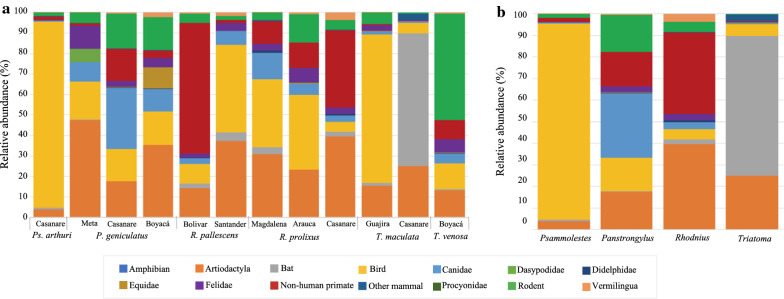
Vertebrate feeding sources depending on geographical location. **a** Relative abundance of feeding sources for each triatomine species and all the regions in which the insects were collected. **b** Relative abundance of feeding sources for each triatomine genus in the department of Casanare. Since the genera *Rhodnius* and *Triatoma* each consisted of two species, we clarify that *R. prolixus* and *T. maculata* are the representatives of their corresponding genus in this case. For both graphs, vertebrate feeding sources are conveniently shown in the arbitrary grouping established for this study

As to whether there were differences among triatomines of the same species in varying geographical locations, for most of the cases, we observed that the read proportions found for each group of vertebrate was variable. Most of the reads corresponding to non-human primates found in *R. pallescens* belonged to individuals sampled in Bolívar; nearly all reads for bats found in *T. maculata* belonged to insects sampled in Casanare; and nearly all reads for Equidae found in *P. geniculatus* belonged to insects sampled in Boyacá, among other cases (Fig. 4a). Also, given that there was one department in which all triatomine genera were sampled, we compared these data and observed that each triatomine genus exhibits different non-human preferences despite the shared location (Fig. 4b), with *Psammolestes* showing a preference for birds, *Triatoma* for bats, *Rhodnius* for artiodactyls and non-human primates, and *Panstrongylus* showing a preference for canids and rodents.

Additionally, since some samples were positive for TcIDom and TcISylv, we evaluated if the presence of this DTU could correspond to vertebrate habitat (e.g. domestic or sylvatic). For the latter, we divided the detected vertebrates in domestic and sylvatic ones (this is shown in Additional file 5: Table S2). We observed that the TcIDom findings did not imply that vertebrates from which the triatomines fed were domestic, given that for every triatomine detected with TcI, the vertebrates composing their diet were both domestic and sylvatic (Additional file 6: Figure S3).

### Statistical analysis

Kruskal-Wallis tests revealed statistically different median values among triatomine species. According to these tests, significantly different medians were found among triatomine species for reads belonging to the Felidae (*χ*^2^ = 11.959, *df* = 5, *P* = 0.035), Didelphidae (*χ*^2^ = 14.558, *df* = 5, *P* = 0.012), Dasypodidae (*χ*^2^ = 12.778, *df* = 5, *P* = 0.026), birds (*χ*^2^ = 12.568, *df* = 5, *P* = 0.028) and bats (*χ*^2^ = 17.277, *df* = 5, *P* = 0.004), which can also be observed graphically (Figs. 2, 3). Exploring the values for these particular vertebrates by performing individual t-tests, we found that *Ps. arthuri* median value for bird reads is significantly different from each of the other triatomine bugs, *T. maculata* median value for the Dasypodidae reads is significantly different from each of the other insects, and that both of these triatomine species have significantly different median values for the Felidae regarding the rest of the triatomines. Also, *P. geniculatus* and *R. prolixus* have significantly different median values for Felidae, and the same happens for *T. venosa* and *P. geniculatus* with bat reads.

**Fig. 5:**
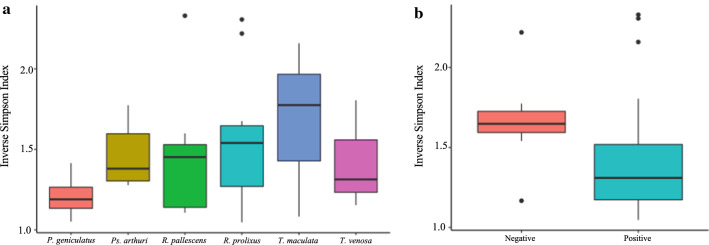
Boxplots displaying inverse Simpson index results for each triatomine genus evaluated (**a**), and for *T. cruzi*-positive and negative samples (**b**)

For the alpha diversity analysis, we calculated the inverse Simpson diversity index for vertebrate species hosts delineated by triatomine species. We found that this index exhibited the highest (1.77) and lowest (1.19) median values for *T. maculata* and *P. geniculatus*, respectively (Fig. 5a), whereas the median value obtained for *Ps. arthuri* was 1.38, 1.45 for *R. pallescens*, 1.54 for *R. prolixus*, and 1.31 for *T. venosa*. Nonetheless, no statistically significant differences were detected when triatomine species were compared with Kruskal-Wallis tests. The overall diversity index of vertebrate species as feeding sources for triatomines was 1.45, which can be considered as being a typical value obtained for this test. We observed a statistical significance in the difference between the diversity index of *T. cruzi*-positive and *T. cruzi*-negative samples (*W* = 188, *P* = 0.02588), where the diversity index was higher for *T. cruzi* negative samples (Fig. 5b).

## Discussion

An understanding of the feeding source patterns for the vectors of human-borne diseases is pivotal for elucidating the relationship of these vectors with their hosts and with the parasites they transmit. To the best of our knowledge, this is the first study to use NGS technologies for several *T. cruzi* vector species to describe their feeding sources in Colombia. Our results suggest that NGS technologies can be used to identify a vast diversity of feeding sources of triatomines. Thus, these technologies could play a crucial role in understanding the ecology of Chagas disease, especially the parasite transmission dynamics, as it has been suggested previously [41].

Herein, we identified 67 animal species as constituents of triatomine feeding habits (Additional file 3: Figure S1, Additional file 4: Figure S2), a higher value than those previously reported [9, 18, 22]. We also found multiple vertebrate feeding sources per triatomine (Figs. 2, 3), which agrees with previous reports assuring that the feeding source of individual insects was not restricted to a single vertebrate host, like recently shown by Dumonteil et al. [9]. Our study identified a greater number of feeding sources than previously reported, possibly because more numerous species were analyzed and larger sample size was used (despite our sample size was relatively low, it was higher than previous reports). We also highlight that the number of reads obtained per sample was around 170,000, which surpasses that reported in Mexico and Colombia [9, 27]. This suggests that read depth should be considered when identifying blood sources across triatomines in order to fully unravel its usefulness within epidemiological studies.

The predominant feeding source detected, found in all triatomines, was *H. sapiens* (Fig. 2), and around 80% of the samples evaluated were infected with *T. cruzi*, which is a similar result to that reported in previous studies on *Triatoma* and *Rhodnius* [42–45]. Human blood was the main feeding source of *R. prolixus* and *P. geniculatus* (Additional file 3: Figure S1), as has been previously reported for these and other triatomine species [8, 9, 31]. Moreover, reads belonging to *H. sapiens* were abundant regardless of the geographical location or the triatomine species, possibly due to the already reported preference of triatomines for the blood of *H. sapiens* [46]. This suggests a highly dynamic transmission in the areas where these insects were collected, which emphasizes the need to intensify prevention and control measures in said areas. A possible explanation for such high abundance of *H. sapiens* reads among the triatomine feeding sources includes the presence of human settlements, even within sylvatic ecotopes throughout the country. Severe deforestation has been reported, and even oral *T. cruzi* transmission outbreaks have occurred in the sampled areas due to human invasion into sylvatic environments [47]. Humans may, therefore, be a common component within the diet of triatomines, even more so than other vertebrates. In our findings, all the triatomine species were found to harbor a remarkable number of reads belonging to *H. sapiens* in their guts (Additional file 3: Figure S1). The previous findings again highlight the importance of these vector species in maintaining the human role in the life-cycle of *T. cruzi* and its importance in public health. Moreover, secondary vectors, such as *T. maculata* and *T. venosa*, should not be discarded, as we also detected reads associated with *H. sapiens* in their guts. In the Andean region, *R. prolixus* has been the focus of vector control programmes, but we herein additionally highlight the need to include other species, particularly *P. geniculatus* and also due to its importance in oral transmission outbreaks in Venezuela and Colombia [48, 49].

Although this is a descriptive study, it is interesting to observe the overall patterns in terms of feeding source diversity (Figs. 2, 3). In most individuals collected in domestic environments, we also found sylvatic-like feeding sources, including non-human primates, artiodactyls, didelphids and chiropterans (Fig. 2). These findings suggest a high dispersal ratio of the studied species, which challenges the current vector control programmes in Colombia. This agrees with previous findings in our country and other countries [8, 9, 50, 51] but contradicts other reports in El Salvador where domestic *T. dimidiata* fed mainly on *H. sapiens*, *C. lupus familiaris* and domestic birds [43].

In the present study, birds were the second most frequent feeding source (Additional file 3: Figure S1), with 10.5% of the total reads detected. This is an interesting finding that shows how challenging it can be to attempt to control the transmission of *T. cruzi*, given that hosts with such a remarkable capacity for dispersion are involved. It is also noteworthy of mention that many of the triatomine species used in this study were once considered to have feeding habits restricted to birds, such as *Ps. arthuri* [52] and *T. maculata* [46]. Since a broader feeding behavior has been observed in the last years [8, 53] as well as in this study, it is possible that the feeding behavior of these triatomine species has suffered changes throughout time (which could have been facilitated by the domiciliation processes they seem to endure [53–55]) or that the detection methods for feeding sources have improved during the last years, allowing researchers to discover an increasingly amount of vertebrates that compose the diet of these insects. Whichever the correct assumption, it is important that this feeding source and its impact on the transmission of the parasite are further studied in the future.

Another interesting observation was finding non-human primates among the most frequent feeding sources (Fig. 3, Additional file 3: Figure S1), implying an interesting scenario in terms of Chagas disease control in the studied regions. It is important to mention that non-human primate genera have been found infected with all DTUs except for TcV and TcBat in various countries within the Americas, thereby incriminating them in the parasite transmission cycle [56–58]. In our results, *R. pallescens*, *P. geniculatus* and *R. prolixus* contained the highest number of reads associated with blood belonging to non-human primates, and this is particularly important due to the ecological landscape of oil plantations in the east of the country (i.e. *Attalea butyracea* and *Elaeis guineensis*). These oil-producing plant forests are highly infested with *R. pallescens*, *P. geniculatus*, and *R. prolixus* and are also frequented by non-human primates. Several studies highlight that these forests containing plants from which oils are derived represent a risk for *T. cruzi* transmission and our findings reinforce this hypothesis [27, 59, 60].

Some trends were identifiable in triatomine species with respect to feeding sources (Fig. 4), supported by the statistically significant difference found in this study. Here, we want to highlight that our aim was to describe the feeding sources and not finding associations. Furthermore, we identified *T. maculata* as the triatomine species that fed mostly on bats. This is an important finding given the importance of the Chiroptera in the cycle of transmission of *T. cruzi* and their usual presence within human dwellings, which can ultimately lead to transmission to humans. Besides, since many bat species have omnivorous feeding habits and can feed on small mammals and triatomines [61, 62] the probability of infection with *T. cruzi* could be slightly higher for these animals and this could translate in more *T. cruzi* transmissions that reach humans.

When evaluated by the Wilcoxon test, only *H. sapiens* showed a statistically significant difference between *T. cruzi-*positive and negative samples (*W* = 64, *P* = 0.04873). This could suggest that the presence of *T. cruzi* in a triatomine can modify the feeding behavior of the bug, but we consider this finding is not enough to reach a conclusion; we therefore encourage further studies focusing in this aspect of the feeding dynamic of triatomines. Despite this, and the absence of significant differences in feeding behavior between these two groups in the rest of vertebrate groups, evaluating differences in feeding sources between *T. cruzi*-positive and *T. cruzi*-negative samples was considered to be worth displaying in this study, as it may prove to be important for our understanding of the eco-epidemiology of Chagas disease since it offers insight into the role of vertebrates within the transmission cycle for both domestic and sylvatic ecotopes, and for this we encourage the development of future studies that further explore this variable. We also found a statistically significant difference between the inverse Simpson diversity index of *T. cruzi*-positive and *T. cruzi*-negative samples (Fig. 5b). The aforementioned highlights the need to detect the presence of *T. cruzi* in feeding-source studies, due to the possibility of identifying vertebrates that could be considered as more relevant in terms of the transmission dynamics of the parasite. Given that the triatomine species did not seem to have an effect in this differentiation, potential behavioral changes in the insect caused by the presence of *T. cruzi* might explain this difference.

Some limitations existed in our study, including a relatively small number of samples, number of regions of the provenance of triatomines, and the fact that the majority of the tested samples were positive for *T. cruzi*, which precluded the possibility of establishing a pattern of *T. cruzi* infections associated with the triatomine diet. It is also worth noting that not all the specimens had the same weight in the overall diet, given that the resulting number of reads detected was different for all of them and the percentages shown throughout this manuscript were calculated in terms of relative abundance (taking the number of reads detected per vertebrate for a sample and dividing them by the total amount of reads of the sample). Additionally, there was no control samples or abundance threshold to evaluate the level of possible cross-contamination and secure that the read diversity detected was indeed an accurate depiction of reality, therefore the blood-source diversity could have been artificially increased and future studies with controls and adequate abundance thresholds are needed. Likewise, a relationship exists between time elapsed and the number of reads capable of being detected, in which the more recently the triatomine has fed, the higher the number of reads that could be obtained. Therefore, considering the transversal nature of this study, not detecting a certain feeding source is not conclusive evidence for a lack of this feeding source within the triatomine diet, but could be a consequence of the amount of time passed since the collection of the insect. Nonetheless, it is important to take into consideration that on certain occasions, it can be problematic to extract intestinal contents from insects, especially when they have been starved for long, which could have been the case in our study, given that we had no information concerning the dietary status of the collected samples [5, 45]. Additionally, given the differences between erythrocyte structure, some feeding sources tend to persist longer in the insect gut, which could alter detected read proportions [63]. We suggest that future studies attempt to overcome these limitations in order to improve the quality of the provided information.

When analyzing the feeding sources at a microgeographical scale, several interesting patterns were depicted in our study (Fig. 4). For example, in the case of *R. pallescens*, insects were collected in Mompox, Bolívar, within the sylvatic ecotope and at two different localities from Santander (i.e. inside houses). The ecological landscape of Bolívar seems complex as individual reads from both domestic and sylvatic vertebrates were detected; however, individuals in Santander, despite presenting the same vertebrate groups, revealed a higher number of reads belonging to domestic vertebrates. The landscape of Bolívar is full of forests, and the draining Magdalena River, as well as the Atlantic Ocean, allows an enzootic cycle as compared with the more urban transmission cycle present in Santander [50]. In recent years, *R. pallescens* has gained importance as vector due to its intrusion into human dwellings and high rates of *T. cruzi* infection [8]. Our findings also suggest the high capacity of this species to adapt and switch from sylvatic to domestic blood preferences. This characteristic is particularly important for defining a good vector within medical entomology [64] and could also explain the lack of association of TcIDom with domestic vertebrates, and TcISylv with sylvatic ones (Additional file 6: Figure S3).

In the case of *R. prolixus* and *P. geniculatus*, human blood was the main feeding source (Additional file 3: Figure S1), as has been previously reported [9, 22, 31]. The ecology of these species is complex as they have been found in armadillo nests as well as within human dwellings [48, 65]. Our findings reinforce the epidemiological importance of *P. geniculatus* in the transmission dynamics of Chagas disease within Colombia. In fact, several oral transmission outbreaks have been linked to this species in Colombia, Venezuela and Brazil [8, 26, 48, 49]. The finding that *P. geniculatus* utilizes several different feeding sources interposes a challenge for Chagas disease vector control in the light of the extreme adaptation this insect may exhibit; in addition, previous reports suggest its conspicuous capability of transmitting TcI, TcII, TcIII and TcIV DTUs [8].

As stated, our methodological approach allowed us to elucidate up to 67 different feeding sources, which, to our knowledge, is the highest reported level of diversity. We calculated the inverse Simpson index per species in terms of reads and observed interesting and particular patterns (Fig. 5a). Despite *T. maculata* showing the highest values for the diversity index, the other triatomine species had similar values, which consequently suggests great adaptation of all triatomine species to different blood sources. Therefore, the triatomine species evaluated here could be considered as insects that maintain the epizootic and enzootic cycles of *T. cruzi*, as previously reported for some of them [27, 66]. These patterns reiterate the great usefulness of identifying blood sources in vectors of infectious diseases caused by parasites, such as *T. cruzi*, in order to understand the ecological behavior and varying adaptation mechanisms. This is one of the greatest advantages of amplicon-based NGS, in which the description of the global diversity of feeding sources within one individual can be elucidated.

In this study, it became apparent that some triatomine interactions have changed over time, and this is important because alterations in these interactions can affect disease transmission. For instance, *T. maculata* was once considered to be one of the triatomine species that fed solely on birds [46], and even though reads belonging to birds were found, this feeding source was not the most abundant one for the triatomine species. More importantly, we found that the diet of this triatomine species also contained domestic vertebrates, suggesting that *T. maculata* could be involved in a domiciliation process, something that had not been previously considered. Of note, *R. pallescens* has been widely associated with palm trees [67]; however, given the presence of *H. sapiens* reads in this bug, it seems likely that this species is capable of intruding within human dwellings and therefore possesses greater mobility than previously thought. Additionally, *Ps. arthuri* were found to feed on humans and were positive for *T. cruzi*, which has also been reported by other authors recently [68] and highlights the urgency of evaluating the vectorial capacity of this triatomine species, which was not considered to be a vector in the past. Interestingly, we found Artiodactyla and Perissodactyla (Equidae) reads in this species, which was unexpected. One possible explanation might be that *Ps. arthuri* might visit different vertebrate settlements or roam in the surroundings occasionally feeding on other vertebrates, which may explain the non-bird feeding sources found in some of these bugs. Also, this NGS approach is much more sensitive than Sanger sequencing, which was used in a previous study from our group [68]. Nevertheless, a larger sample size is needed to fully understand the transmission dynamics in *Ps. arthuri.*

## Conclusions

To our knowledge, this is the first study to employ amplicon-based NGS of the *12S* rRNA region to depict blood-feeding sources of various triatomine species collected in different regions of Colombia. Our findings report a striking diversity of blood-feeding sources that had never been previously reported. Despite being a mainly descriptive study, we highlight the generalist behavior of the insects evaluated, and the differences existing among the diets of triatomine species. Consequently, and considering that our methodology does not impose a threat to the studied vertebrates, we propose the performance of similar studies to examine new regions considered as non-endemic for Chagas disease and to profoundly investigate triatomine interactions with possible hosts with the intention of improving control strategies for this disease in Colombia.

## Supplementary information

**Additional file 1: Table S1.** Complete dataset of the 42 triatomines used in this study. This dataset contains ID code, triatomine species, qPCR result, DTU information obtained through conventional PCR, geographical information (region and town), and correlating information for the insect (ecotope and sex).

**Additional file 2: Alignment S1.** FASTA file with the 543 vertebrate species used as reference in this study.

**Additional file 3: Figure S1.** Relative abundance of the 15 vertebrate arbitrary groups within each collected triatomine species.

**Additional file 4: Figure S2.** Relative abundance of reads corresponding to each triatomine species, regarding their division among the 15 arbitrary vertebrate groups.

**Additional file 5: Table S2.** Arbitrary grouping of the vertebrate species. The 67 vertebrate species detected were grouped in 15 different categories, which are used consistently through this study. This file also displays which categories were considered as domestic or sylvatic when this division was considered necessary for the analysis: an asterisk (*) indicates the groups with domestic species, while a plus (+) indicates the groups with sylvatic species. If the vertebrate group was considered to contain both domestic and sylvatic species, the asterisk was placed next to the species considered as domestic, understanding from this that the unmarked species are considered sylvatic.

**Additional file 6: Figure S3.** Number of reads found for each type of TcI DTU (Dom, Sylv and Dom-Sylv). Bar colors represent the habitat of the detected vertebrate (i.e. domestic or sylvatic).

## Data Availability

The data supporting the conclusions of this article are included within the article and its additional files. The dataset generated during the present study was deposited at DDBJ/ENA/GenBank under the study accession number: PRJEB38830.

## Abbreviations

NTD: neglected tropical disease
DTU: discrete typing unit
NGS: next-generation sequencing
PCR: polymerase chain reaction
qPCR: quantitative (real-time) polymerase chain reaction
rRNA: ribosomal ribonucleic acid
QC: quality control
QIIME: quantitative insights into microbial ecology
NCBI: National Center for Biotechnology Information
nd: not detected
