# Supplementary figures and images for: Identification of blood-feeding sources in Panstrongylus, Psammolestes, Rhodnius and Triatoma using amplicon-based next-generation sequencing
Source: Parasit Vectors. 2020 Aug 31;13:434. doi: 10.1186/s13071-020-04310-z (PMC7457505; doi:10.1186/s13071-020-04310-z)

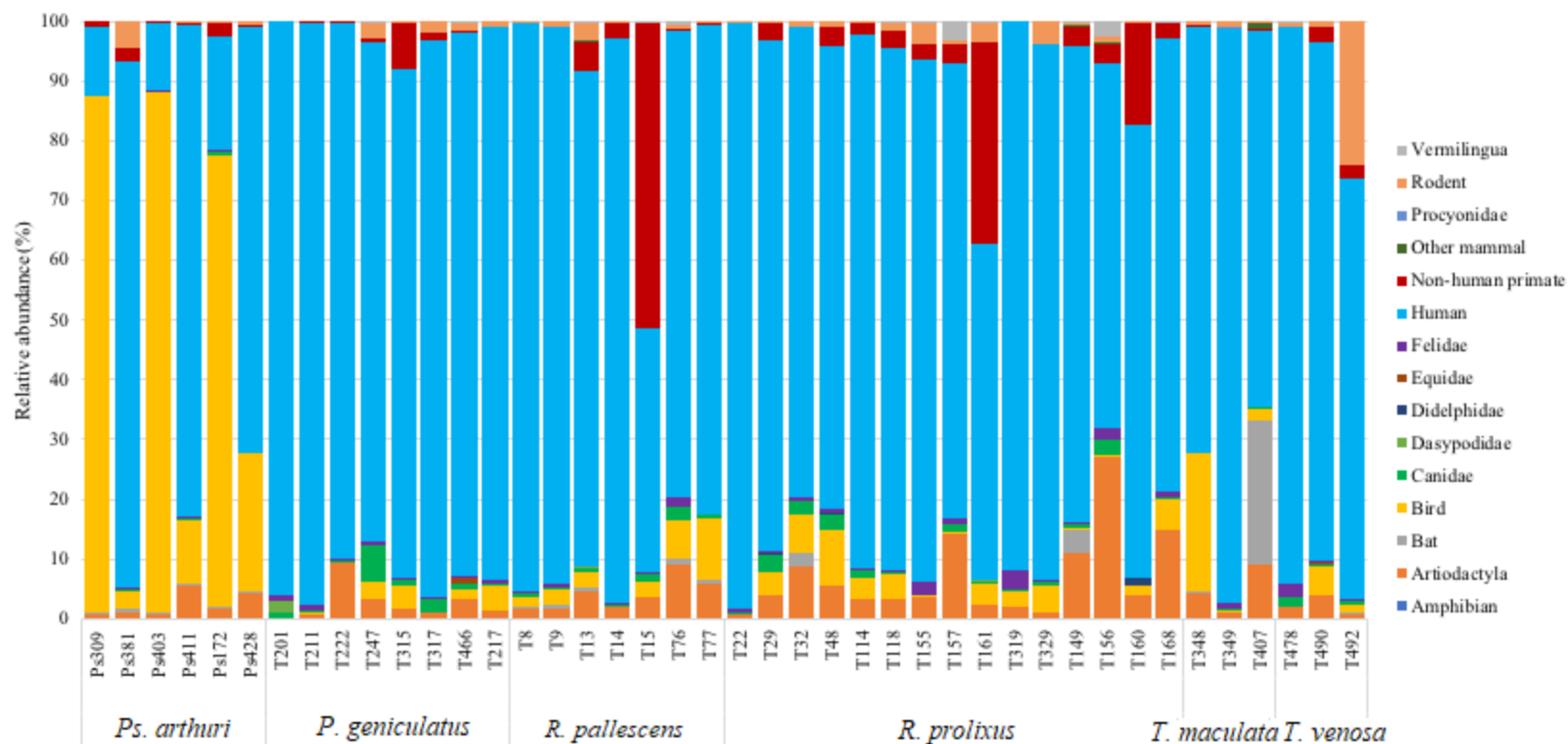

Supplement: Supplementary file 3 — Additional file 3: Figure S1. Relative abundance of the 15 vertebrate arbitrary groups within each collected triatomine species. [file 13071_2020_4310_MOESM3_ESM.pdf]

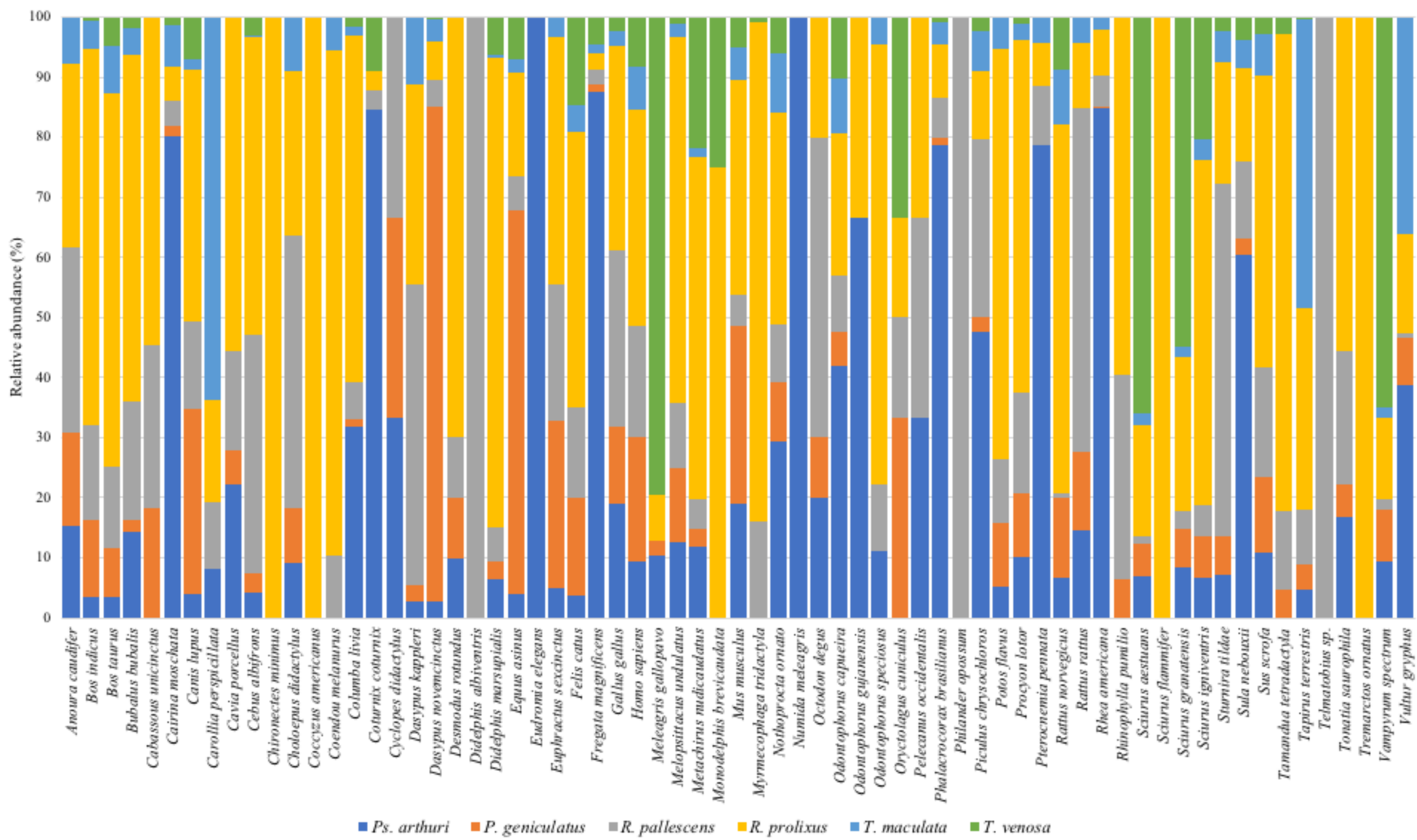

Supplement: Supplementary file 5 — Additional file 5: Table S2. Arbitrary grouping of the vertebrate species. The 67 vertebrate species detected were grouped in 15 different categories, which are used consistently through this study. This file also displays which categories were considered as domestic or sylvatic when this division was considered necessary for the analysis: an asterisk (*) indicates the groups with domestic species, while a plus (+) indicates the groups with sylvatic species. If the vertebrate group was considered to contain both domestic and sylvatic species, the asterisk was placed next to the species considered as domestic, understanding from this that the unmarked species are considered sylvatic. [file 13071_2020_4310_MOESM5_ESM.pdf]

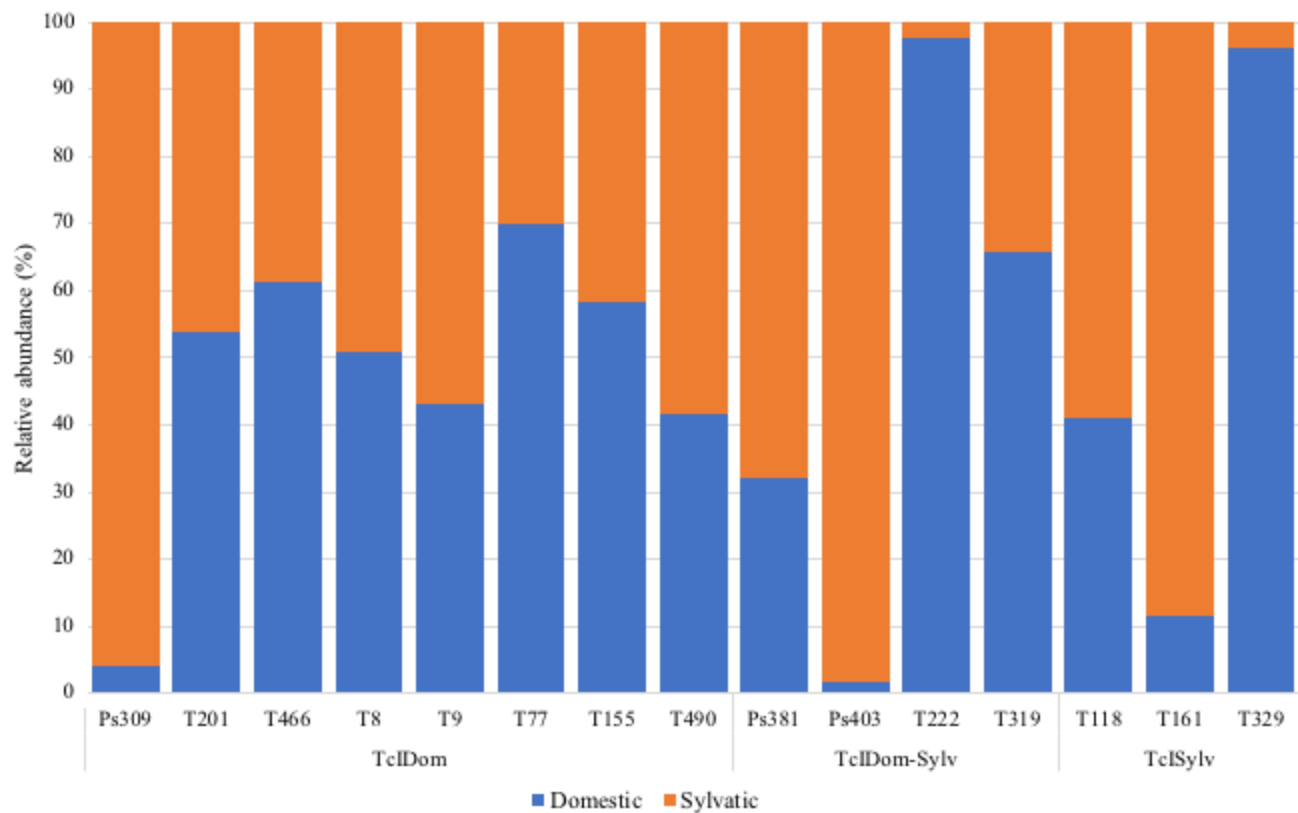

Supplement: Supplementary file 6 — Additional file 6: Figure S3. Number of reads found for each type of TcI DTU (Dom, Sylv and Dom-Sylv). Bar colors represent the habitat of the detected vertebrate (i.e. domestic or sylvatic). [file 13071_2020_4310_MOESM6_ESM.pdf]
